# Antimicrobial Sales Comparison before and after the Implementation of Nationwide Restriction Policy in Saudi Arabia

**DOI:** 10.3390/antibiotics13010015

**Published:** 2023-12-21

**Authors:** Sulaiman M. Alajel, Khaloud O. Alzahrani, Amal A. Almohisen, Meshael M. Alrasheed, Salwa M. Almomen

**Affiliations:** 1 Reference Laboratory for Microbiology, Executive Department of Reference Laboratories, Research and Laboratories Sector, Saudi Food and Drug Authority (SFDA), Riyadh 11561, Saudi Arabia; 2 Molecular Biology Division, Reference Laboratory for Microbiology, Executive Department of Reference Laboratories, Research and Laboratories Sector, Saudi Food and Drug Authority (SFDA), Riyadh 11561, Saudi Arabia; kozahrani@sfda.gov.sa; 3 Statistics Department, King Saud University, Riyadh 11451, Saudi Arabia; 4 Drug Safety and Risk Management, Drug Sector, Saudi Food and Drug Authority (SFDA), Riyadh 13513, Saudi Arabia; 5 Research and Studies Department, Research and Laboratory Sector, Saudi Food and Drug Authority (SFDA), Riyadh 13513, Saudi Arabia

**Keywords:** IQVIA, AMR, AWaRe, pharmaceutical policy, antimicrobial stewardship, antibiotic sales, policy impact evaluation

## Abstract

Antimicrobial dispensing without a prescription has been identified as a significant contributor to the burgeoning crisis of antimicrobial resistance. To combat this, the Saudi Ministry of Health introduced a stringent antimicrobial restriction policy in mid-2018, mandating prescriptions for all antimicrobial drug dispensations at pharmacies. Therefore, this study aimed to assess the immediate impact of this policy on retail antimicrobial sales. To do so, we analyzed annual sales data from 2017 to 2019 sourced from the IQVIA-MIDAS^®^ database, which included a range of antimicrobials, such as antibiotics, antifungals, and other related agents. The analysis revealed a notable reduction in overall antimicrobial sales by 23.2%, decreasing from 818.9 million SAR in 2017 to 648.4 million SAR in 2019. While the Wilcoxon signed-rank test indicated a statistically significant median reduction in total antimicrobial sales post-policy implementation (*p* = 0.0397), it is important to acknowledge that the long-term effects and adherence to the policy require further investigation. Notably, sales of amoxicillin dropped by 70% in 2019 compared to 2017, contributing largely to the decline. Conversely, a continuous increase in sales of some antimicrobial drugs following the restriction policy was observed, led by amoxicillin/clavulanic acid. Our data support the implementation of antimicrobial restriction measures as an effective means of controlling excessive antimicrobial sales and dispensing without prescriptions.

## 1. Introduction

Antimicrobial resistance (AMR) is a global healthcare issue that raises mortality from infectious diseases and increases healthcare costs [1,2]. According to the World Health Organization (WHO), AMR will become the leading cause of death worldwide by 2050 unless addressed properly [3]. Coupled with this, the emergence of multidrug-resistant (MDR) pathogens poses a significant threat to global health.

Recent reports found that about 33,000 individuals die in the European Union each year because of infections caused by AMR. Notably, 39% of these fatalities are associated with bacteria resistant to last-resort medicines, such as carbapenems and colistin [4]. In the United States, a parallel scenario exists, with an estimated 2.8 million individuals infected with antibiotic-resistant bacteria each year, leading to over 35,000 fatalities [5]. The impact of AMR extends beyond health to the economy, with the cost of treatment involving expensive, last-line antimicrobials and extended hospital stays ranging from 6.4 to 12.7 additional days, placing a significant burden on healthcare systems [6]. For example, the economic burden caused by infection with AMR bacteria reached an estimated EUR 1.5 billion annually in the EU and USD 55 billion in the U.S. [4]. Unfortunately, mortality and economic burden data caused by AMR in Saudi Arabia have been poorly measured, highlighting a critical need for national surveillance programs to assess and address this issue [7].

A comprehensive recent study of human antimicrobials consumption, measured in defined daily use (DDD), showed an increase of 65% in consumption between the years 2000 and 2015, especially among the last-resort antibiotics, such as glycylcyclines, oxazolidinones, carbapenems, and polymyxins [8]. This uptick in usage has been pronounced mostly in low- and middle-income countries, paralleled by a worrying decline in the development of novel antimicrobials and the rise of multidrug-resistant pathogens. Therefore, more attention must be given to prescribing policies to control antimicrobial misuse and maximize the life expectancy of a given drug. Numerous studies have confirmed a positive correlation between antimicrobial consumption and the increased emergence of AMR worldwide [9,10,11,12,13,14,15]. The elevation in AMR levels can be attributed to several factors, including inadequate therapy duration, incorrect dosages, and irrational fixed-dose drug combinations, but the most predominant cause is the improper use of antimicrobials [16].

The dispensing of antimicrobials without a prescription (DAwP) for self-limiting conditions, such as upper respiratory tract infections (URTIs) as well as urinary tract infections (UTIs), is a key driver in the emergence and spread of AMR. In the past, regulations against the sale of over-the-counter (OTC) antimicrobials were not strictly enforced in some countries, leading to a prevalent use of non-prescribed antimicrobials for self-limiting conditions, such as the common cold [17]. Van Boeckel et al. analyzed national pharmaceutical sales data for 71 countries around the globe for the years 2000 to 2010 to examine variations in consumption in different countries [18]. This study highlighted the wide variation in antimicrobial use across countries, with some countries having significantly higher rates of consumption. 

In Saudi Arabia, the prohibition of OTC antimicrobial sales in private pharmacies was not enforced, and non-prescribed use of antimicrobials was common [17]. Several studies conducted across Saudi Arabia over the past years have shown that DAwP is common among pharmacists. For example, Al-Ghamdi showed that 82.0% of 88 community pharmacies in the Eastern Province of Saudi Arabia visited by simulated clients presenting cases of uncomplicated UTIs were dispensed antimicrobials without prescription [19]. Emeka et al. found a high incidence of self-medication, where colds and sore throats were the most common reasons for seeking DAwP [20]. Bin Abdulhak et al. highlighted a high incidence of non-prescribed sales of antimicrobials across pharmacies in the Riyadh region [21]. Another study conducted by Al-Qahtani et al. revealed the high prevalence of self-medication with antimicrobials among patients attending primary care clinics in King Khalid University Hospital, Riyadh, Saudi Arabia. According to this study, the main reasons for self-medication included mild illness, prior experience with similar symptoms, and perceived knowledge of the appropriate treatment [22]. A more recent study showed that 70.7% of community pharmacists in Saudi Arabia reported that DAwP was common among community pharmacists [23].

The pressing issue of AMR has prompted urgent calls led by the WHO to address this issue, including the development of international and country-specific intervention plans in both community and hospital settings to control antimicrobial usage in order to decrease AMR levels [24]. Therefore, global stewardship programs and governmental policies were implemented in many countries to reduce overall antibiotic prescribing and, hence, reduce the burden of resistance [8,18,25,26,27]. In response to that, the Saudi MOH initiated a nationwide antibiotic restriction policy in mid-2018, in which pharmacies are strictly prohibited from dispensing antibiotics without physicians’ prescriptions. Failure to adhere to the regulations may result in high penalties for the license owner, including cancellation of the license, a fine of up to 100 thousand Saudi Arabian Riyals (SAR), and imprisonment for up to six months [28]. Moreover, many educational approaches were implemented by the Saudi MOH with a focus on changing the practice of healthcare providers and increasing public awareness of the importance of rationalization and optimal use of antibiotics to reduce the overuse and misuse of antibiotics. Other approaches were also implemented by the Saudi MOH, including policies such as professional regulation, restricted reimbursement, and prescription requirements. Unfortunately, the effectiveness of the Saudi MOH’s restrictive measures on antimicrobial sales has not been rigorously assessed. Therefore, the objective of this study aimed to assess the immediate impact of the MOH policy on retail antimicrobial sales. Understanding the outcomes of governmental policies is vital for informing policymakers and refining future intervention measures to combat AMR effectively.

## 2. Results

In this study, a total of 38 antimicrobials were extracted from the IQVIA database, including 25 antibiotics belonging to different classes (penicillins; beta-lactam/beta-lactamase inhibitors; macrolides; first-, second-, and third-generation cephalosporins; quinolones/fluoroquinolones; tetracycline; and oxazolidinones), 5 antifungals, 3 antimycobacterials, 4 combinations, and 1 classified as “other” (Table 1). Amoxicillin/clavulanic acid was the most commonly sold drug in both the pre-intervention policy (2017) and post-intervention policy (2019) periods. The most sold drugs in the pre-intervention policy period were amoxicillin, ciprofloxacin, azithromycin, cefixime, sulfamethoxazole/trimethoprim, cefuroxime axetil, and cefalexin. After the implementation of the restriction policy, the total sales of antimicrobials decreased by 23.2%, from SAR 818,916,437 in 2017 to SAR 648,383,630 in 2019. The nonparametric Wilcoxon signed-rank test was applied to the overall antimicrobial sales before and after restriction. This test showed that there was a statistically significant decrease in antimicrobial median sales after the intervention in 2019 compared to sales before the intervention in 2017, with a *p*-value of 0.0397 (Table 2). Twenty-four out of 38 (63.2%) antimicrobials showed a decline in sales after the restriction, where sales were 420,123,138 SAR before the restriction and decreased to 186,143,367 SAR after the restriction. Out of these 24 antimicrobials, 12 were classified as “Watch” according to WHO AWaRe classification, 7 were classified as “Access”, 1 as “Reserve”, and one was unclassified. On the contrary, 14 out of the 38 antimicrobials (36.8%) showed an increase in sales from SAR 398,793,299 in 2017 to SAR 462,240,263 SAR in 2019. Out of these 14 antimicrobials, 8 were classified as “Watch”, 2 as “Access”, 1 as “Reserved”, and 3 were “unclassified”. Antibiotics penicillin (amoxicillin) and combinations of penicillin with ß-lactamase inhibitors (amoxicillin/clavulanic) accounted for more than 50% of the total procurement sales for all drugs, yet the restriction was effective only in reducing penicillin (amoxicillin) sales by 70%, from SAR 208,562,130 before intervention in 2017 to SAR 61,968,664 SAR after intervention in 2019. Unlike penicillin, the intervention effects were limited to the sales of combinations of penicillin with ß-lactamase inhibitors (amoxicillin-clavulanic acid), with an increase in sales of SAR 17,673,993 (6.5%) in 2019 compared to 2017. All antimicrobial sales information during pre- and post-intervention is listed in Table 1.

## 3. Discussion

The main objective of this study was to investigate the impact of the MOH regulation on antimicrobial sales in Saudi Arabia by comparing sales before and after the implementation of restrictive measures. Our data demonstrated a notable reduction in total sales of antimicrobials in 2019 compared to sales in 2017. This decrease in antimicrobial sales following the restriction policy implemented in 2018 suggested that this decrease might be associated with successful MOH intervention. This comes in agreement with previous studies that showed a direct reduction in antimicrobial sales after policy intervention in many countries around the world, such as Lantin American countries, Italy, India, France, and China [29,30,31,32,33,34,35,36,37,38,39].

Notably, we observed a significant decline in sales of amoxicillin—one of the top-selling antibiotics in Saudi Arabia—with a 70% decrease in 2019 compared to 2017. This suggests that the inappropriate use of penicillin may have diminished after the restrictions were enforced. In contrast, sales of amoxicillin/clavulanate (broad-spectrum antibiotics) increased by 6.5% in 2019 compared to 2017. This one drug was responsible for the general increase in total antimicrobial sales as it holds the highest sales. Similar to our study, a previous study assessed the impact of restricting OTC sales of antimicrobial drugs in Brazil and showed a reduction in the number of units sold for all penicillins except amoxicillin in combination with clavulanic [31]. Subsequent research conducted between 2008 and 2012 in both Brazil and Mexico observed a notable decline in penicillin sales in Brazil, while there was an increase in the sales of other antibiotics in Mexico [29,32]. For future directions, it is critical to maintain rigorous control of antibiotic dispensation policies and to strengthen educational campaigns targeting both healthcare professionals and the general public regarding the appropriate use of antibiotics. Furthermore, investing in obtaining and developing quick diagnostic technologies may optimize antibiotic selection, assuring targeted therapy that will decrease AMR while preserving the efficacy of broad-spectrum antibiotics, such as amoxicillin/clavulanate. These efforts will be critical in sustaining the gains achieved through the implemented restrictive measures and in promoting a stronger response to antibiotic resistance trends.

Our results also showed a continuous increase in sales for some other antimicrobials in the pre-intervention policy period (Table 1). Unfortunately, most of these antimicrobials belong to antibiotics that are classified as “Watch” according to the WHO classification system. This was not expected given the restrictive policy implemented by MOH and governmental potential fines, indicating a relatively inadequate prescribing practice. This is in line with a global survey that revealed amoxicillin/clavulanate was the most commonly sold antibiotic in 75 countries and represented nearly 50% of sales [25]. Our study also revealed the increase in sales of certain broad-spectrum antibiotics after the intervention, including azithromycin, clarithromycin, cefalexin, cefdinir, levofloxacin, and moxifloxacin. These drugs are used to treat bacterial infections in many parts of the human body and are all classified as “Watch” antibiotics. The rise aligns with previous findings that showed that the percentage of broad-spectrum antibiotic use did not achieve a significant decline following intervention measures [32]. Indeed, a shift from the use of narrow-spectrum antibiotics towards broad-spectrum antibiotics after intervention was observed in several European countries [40]. Comparable patterns have been noted in China despite the introduction of the 2011 national antimicrobial stewardship campaign [36]. A similar situation was observed in Korea following the adoption of antimicrobial stewardship programs in Korean tertiary hospitals [41]. Moreover, there has been a notable increase in the prescription of broad-spectrum antibiotics, coupled with a decrease in the use of narrow-spectrum ones in Denmark [42]. 

Unfortunately, the misuse of broad-spectrum antibiotics has contributed tremendously to the development of multidrug-resistant pathogens, unlike the targeted use of narrow-spectrum antibiotics. This continuous growth in some broad-spectrum antibiotic sales, even after restriction measures have been implemented, may be attributed to the fact that this class of antibiotics is frequently inappropriately prescribed, even in cases when narrow-spectrum counterparts may be more suitable. A study that used data from the European Surveillance of Antimicrobial Consumption to establish reliable quality indicators for outpatient antibiotic usage in Europe showed that consistently high or rising trends in the use of broad-spectrum penicillins are generally indicative of poor medical practices [40]. This can often be attributed to the common tendency of physicians to broaden empiric antibacterial therapy before the microbiological test results are known, which may elevate the difficulty of reducing broad-spectrum antibiotic sales and usage. Therefore, primary care physicians and their patients should be potential targets for intervention guidelines in daily clinical practice, especially in the private sector. Other causes can include the ease of once-daily administration with some broad-spectrum drugs or perhaps their accessibility or affordable price. However, further research is needed to explore other factors, such as economic and population growth factors, that may be contributing to the changes in antimicrobial sales in this country.

To the best of our knowledge, the present study is one of very few that offers Saudi policymakers with validated outcomes on specific governmental interventions addressing previous uncontrolled antimicrobial sales. However, several limitations of this study need to be addressed. First, the IQVIA database does not distinguish sales of prescription from non-prescription antimicrobials; therefore, drug sales do not accurately reflect consumption patterns in Saudi Arabia. Second, the IQVIA database includes all antimicrobial sales from both the private and public sectors. Therefore, changes in sales after the restriction can have a greater impact on the private sector, given that in the public sector, a prescription is needed to get medicines even before the regulation. Third, the observed reduction in total antimicrobial sales in our study can be attributed to other factors besides the restriction policy, such as socioeconomic factors, government inspections, prescribing practices, and seasonality effects. Follow-up investigations in subsequent years are required to confirm if this decline in sales is a rather true effect of the restriction than an isolated incident. Finally, we acknowledge that our results may not reflect the full picture of the restriction policy impact on antimicrobial use in Saudi Arabia, but they give an indication of the governmental intervention effect. Despite its limitations, this study may be considered a baseline for further studies to evaluate the effectiveness of MOH interventions. Since the MOH implementations were recently applied in Saudi Arabia, further studies must be conducted to evaluate whether these policies have changed the profile of antibiotic use and the antimicrobial resistance rate.

## 4. Materials and Methods

### 4.1. Study Design

This is a retrospective study that involved assessing antimicrobial sales one year before and after the initiation of the nationwide restriction program in Saudi Arabia in mid-2018. All antimicrobial drugs, such as antibiotics, antifungals, antimycobacterials, and others, were included regardless of brand or generic name or route of administration. Antimicrobial drugs that had missing data before or after restriction policy implementation were excluded from this study.

### 4.2. Data Collection

The main outcome of interest for this study was to determine if the antimicrobial restriction policy implemented by the Saudi MOH in 2018 influenced the sales of antimicrobials, encompassing both potential decreases and increases. To do so, antimicrobial sales data during the pre-implementation phase of the sales restriction policy in 2017 and the post-implementation phase in 2019 were extracted from the IQVIA-Multinational Integrated Data Analysis System (IQVIA-MIDAS^®^) for comparison. The year 2018 was not included in the comparison. This is because this year is when the implementation phase took place, and usually, the effectiveness of such a policy can take up to 3–6 months to achieve its goals. IQVIA-MIDAS^®^ is an international commercial database company that tracks pharmaceutical sales by collecting data from manufacturers, distributors, and wholesalers through retail and non-retail channels. This includes OTC and prescription-based antimicrobial sales. In the case of Saudi Arabia, this IQVIA database comprises 98% retail channels and 85% public channels actual sales, and data coverage is projected to cover 100% of all sales information in the near future [43]. Antibiotics were categorized according to the WHO (AWaRe) classification (Access, Watch, Reserve) or unclassified for antifungals and antimycobacterials [44]. The full list of antimicrobial drugs and classes is included in Table 1.

### 4.3. Statistical Analysis

The normality of the differences in the antimicrobial sales data was tested using the Kolmogorov–Smirnov normality test [45]. Since the antimicrobial sales data were not normally distributed but rather skewed towards specific drugs, a nonparametric Wilcoxon signed-rank test for paired samples was used. Here, we applied this test to the overall antimicrobial sales to compare intervention outcomes between the pre-intervention sales period (2017) and the post-intervention sales period (2019). The statistical significance was set at *p*  <  0.05. Analysis was conducted with R Version 3.6.3.

## 5. Conclusions

This study examined the impact of the Saudi MOH’s antimicrobial restriction policy on the sales of antimicrobials in Saudi Arabia. Our analysis, focusing on the periods before and after the policy implementation, revealed a significant reduction in the overall sales of antimicrobials. This decrease was predominantly evident in the reduced sales of antibiotics such as amoxicillin, suggesting a shift in prescribing patterns possibly influenced by the policy. However, the study also identified an increase in the sales of certain antibiotics, particularly amoxicillin/clavulanic acid. This observation underscores the complex dynamics of antimicrobial sales and the need for continuous monitoring and tailored strategies to mitigate any unintended consequences of policy changes. The results highlight the effectiveness of stringent sales restriction policies in curbing the overall antimicrobial sales, thus potentially contributing to the global effort against antimicrobial resistance (AMR). Nevertheless, the study also points to the necessity for ongoing surveillance and refinement of such policies. The variation in the impact across different antimicrobial drug classes suggests that, while restrictions can lead to overall reductions in sales, specific classes may require more targeted interventions. This study serves as a crucial baseline for further research and policy development in Saudi Arabia and offers insights that could be valuable for other regions implementing similar restriction policies. Future research efforts should focus on a more granular analysis of antimicrobial sales trends, including the evaluation of the impact on prescription practices, antibiotic use, and the potential effects on AMR patterns.

## Figures and Tables

**Table 1:** Sales of antimicrobial drugs in Saudi Arabia comparing pre- and post-intervention policy periods.

Antimicrobials	Drugs	WHO AWaRe Classification	Pre-intervention SAR (%)	Post-intervention SAR (%)	Difference (SAR)
Penicillin	Amoxicillin	Access	208,562,130 (25.5)	61,968,664 (9.1)	↓ 146,593,466
Penicillin/Beta-lactamase Inhibitor	Amoxicillin + Clavulanic Acid	Access	271,364,372 (33.1)	289,038,365 (44.6)	↑ 17,673,993
Macrolides	Azithromycin	Watch	39,095,341 (4.8)	43,139,002 (6.7)	↑ 4,043,661
	Erythromycin	Watch	914,020 (0.1)	539,240 (0.08)	↓ 374,780
	Clarithromycin	Watch	18,545,561 (2.3)	23,334,466 (3.6)	↑ 4,788,905
First-generation Cephalosporin	Cefradine	Access	1,677,600 (0.2)	479,020 (0.07)	↓ 1,198,580
	Cefadroxil	Access	6,600,452 (0.8)	3,462,156 (0.5)	↓ 3,138,296
Second-generation Cephalosporin	Cefalexin	Access	21,629,340 (2.6)	46,156,260 (7.1)	↑ 24,526,920
	Cefuroxime Axetil	Watch	27,796,270 (3.4)	31,428,750 (4.8)	↑ 3,632,480
	Cefprozil	Watch	3,744,210 (0.5)	1,089,560 (0.2)	↓ 2,654,650
Third-generation Cephalosporin	Cefaclor	Watch	18,285,024 (2.2)	11,757,886 (1.8)	↓ 6,527,138
	Cefdinir	Watch	5,679,308 (0.7)	9,693,604 (1.5)	↑4,014,296
	Cefpodoxime Proxetil	Watch	10,213,588 (1.2)	5,682,064 (0.9)	↓ 4,531,524
	Cefditoren Pivoxil	Watch	3,169,480 (0.4)	22,600 (0.1)	↓ 3,146,880
Fluoroquinolones	Cefixime	Watch	34,529,250 (4.2)	27,772,900 (4.0)	↓ 6,756,350
	Ciprofloxacin	Watch	40,272,280 (4.9)	27,376,890 (4.3)	↓ 12,895,390
	Gemifloxacin	Watch	1,610,822 (0.2)	868,392 (0.1)	↓ 742,430
	Levofloxacin	Watch	5,645,563 (0.7)	7,476,202 (1.2)	↑ 1,830,639
	Moxifloxacin	Watch	3,162,084 (0.4)	4,127,579 (0.6)	↑ 965,495
	Ofloxacin	Watch	1,996,730 (0.2)	1,122,960 (0.1)	↓ 873,770
	Norfloxacin	Watch	632,212 (0.1)	280,448 (0.04)	↓ 351,764
Tetracyclines	Doxycycline	Access	19,946,290 (2.4)	12,218,450 (1.9)	↓ 7,727,840
	Minocycline	Reserve	7,150,100 (0.9)	1,989,260 (0.3)	↓ 5,160,840
Oxazolidinones	Clindamycin	Access	5,412,908 (0.7)	4,439,158 (0.7)	↓ 973,750
	Linezolid	Reserve	141,760 (0.02)	176,130 (0.03)	↑ 34,370
Antifungals	Fluconazole	Unclassified	2,094,186 (0.3)	1,762,070 (0.3)	↓ 332,116
	Voriconazole	Unclassified	182,086 (0.02)	232,552 (0.04)	↑ 50,466
	Itraconazole	Unclassified	2,407,586 (0.3)	2,790,061 (0.4)	↑ 382,475
	Posaconazole	Unclassified	15,183 (0.0)	252 (0.0)	↓ 14,931
	Terbinafine	Unclassified	1,160,128 (0.1)	1,490,004 (0.2)	↑ 329,876
Antimycobacterial	Nystatin	Unclassified	1,137,054 (0.1)	8,700 (0.0)	↓ 1,128,354
	Isoniazid	Unclassified	791,939 (0.1)	201,733 (0.03)	↓ 590,206
	Rifampicin	Watch	1,022,716 (0.1)	1,864,984 (0.3)	↑ 842,268
Others	Isoniazid + Rifampicin	Watch	961,184 (0.1)	1,292,304 (0.2)	↑ 331,120
	Sulfamethoxazole + Trimethoprim	Access	32,983,940 (4.0)	10,580,290 (1.6)	↓ 22,403,650
	Ciprofloxacin + Phenazopyridine	Watch	736,100 (0.1)	136,610 (0.02)	↓ 599,490
	Cefuroxime Axetil	Watch	3,047,100 (0.4)	478,480 (0.1)	↓ 2,568,620
	Metronidazole	Access	14,600,540 (1.8)	11,905,584 (1.8)	↓ 2,694,956
Total			818,916,437	648,383,630	↓ 170,532,807

**Table 2:** Wilcoxon signed-rank test to compare differences in antimicrobials sales from 2017 (pre-intervention period) to 2019 (post-intervention period). The statistical significance was set at *p* < 0.05.

	Median	IQR	*p*-Value
**Pre-intervention Sales (SAR)**	3,456,845	17,337,604	0.0396
**Post-intervention Sales (SAR)**	2,389,661	11,374,585	

## Data Availability

The dataset that supports the findings of this study is available from IQVIA™ Saudi Arabia.
